# Predicting outcomes in persistent atrial fibrillation: the impact of surface ECG f-wave amplitude following pulmonary vein isolation

**DOI:** 10.1007/s10840-025-02018-7

**Published:** 2025-02-19

**Authors:** Aruran Baskaralingam, Matteo Marchetti, Jorge Solana-Munoz, Cheryl Teres, Mathieu Le Bloa, Alessandra Pia Porretta, Giulia Domenichini, Ciro Ascione, Laurent Roten, Sven Knecht, Michael Kühne, Christian Sticherling, Patrizio Pascale, Etienne Pruvot, Adrian Luca

**Affiliations:** 1 Service of Cardiology, Lausanne University Hospital, University of Lausanne, Rue du Bugnon 46, CH-1011 Lausanne, Vaud Switzerland; 2 Service of Cardiology, APHP Hôpital Bichat, Paris, France; 3 Department of Cardiology, Inselspital, Bern University Hospital, University of Bern, Bern, Switzerland; 4 Department of Cardiology, University Hospital of Basel, Basel, Switzerland

**Keywords:** Atrial fibrillation, Pulmonary vein isolation, F-wave amplitude, Surface electrocardiogram

## Abstract

**Background:**

Fibrillatory wave amplitude (fWA) on 12-lead ECG predicts the outcome of ablation in atrial fibrillation (AF). We hypothesized that changes in fWA following wide circumferential isolation of pulmonary veins (WPVI) in persistent AF (peAF) is a better predictor of ablation outcome compared to baseline fWA.

**Methods:**

Eighty-nine patients (sustained peAF 7 ± 7 months) underwent a first-time WPVI. Sixty-second ECG signals devoid of QRST waves were recorded at baseline and at the end of the WPVI (endWPVI). fWA for each ECG lead and mean fWA (meanfWA) across the 12-lead ECG were computed. Patients with recurrence after the index WPVI underwent a redo to ensure complete PVI. The primary endpoint was long-term AF freedom OFF antiarrhythmics drugs (AADs) after one or two WPVI (SUCCESS group). The FAILURE group was defined as AF recurrence post-redo.

**Results:**

Over a mean follow-up of 35 ± 10 months, freedom from AF OFF AADs was achieved in 61% (SUCCESS group), while 29% had AF recurrence after redo WPVI (FAILURE group). The SUCCESS group showed significantly higher fWA values in ECG leads V_1_, V_4_, and V_5_ at baseline (*p* < 0.05), as well as in leads III, aVL, aVF, and V_4_, and in meanfWA at endWPVI (*p* < 0.05) compared to the FAILURE group. A baseline mean fWA ≥ 0.044 mV or a decrease in mean fWA ≤ 11% following WPVI predicted long-term sinus rhythm restoration with a sensitivity of 81% and a specificity of 69% (*p* < 0.05).

**Conclusion:**

Low fWA values and a significant reduction in fWA following WPVI are associated with a high risk of AF recurrence in patients with peAF.

**Graphical Abstract:**

Fibrillatory wave amplitude changes after WPVI can predict long-term sinus rhythm maintenance.

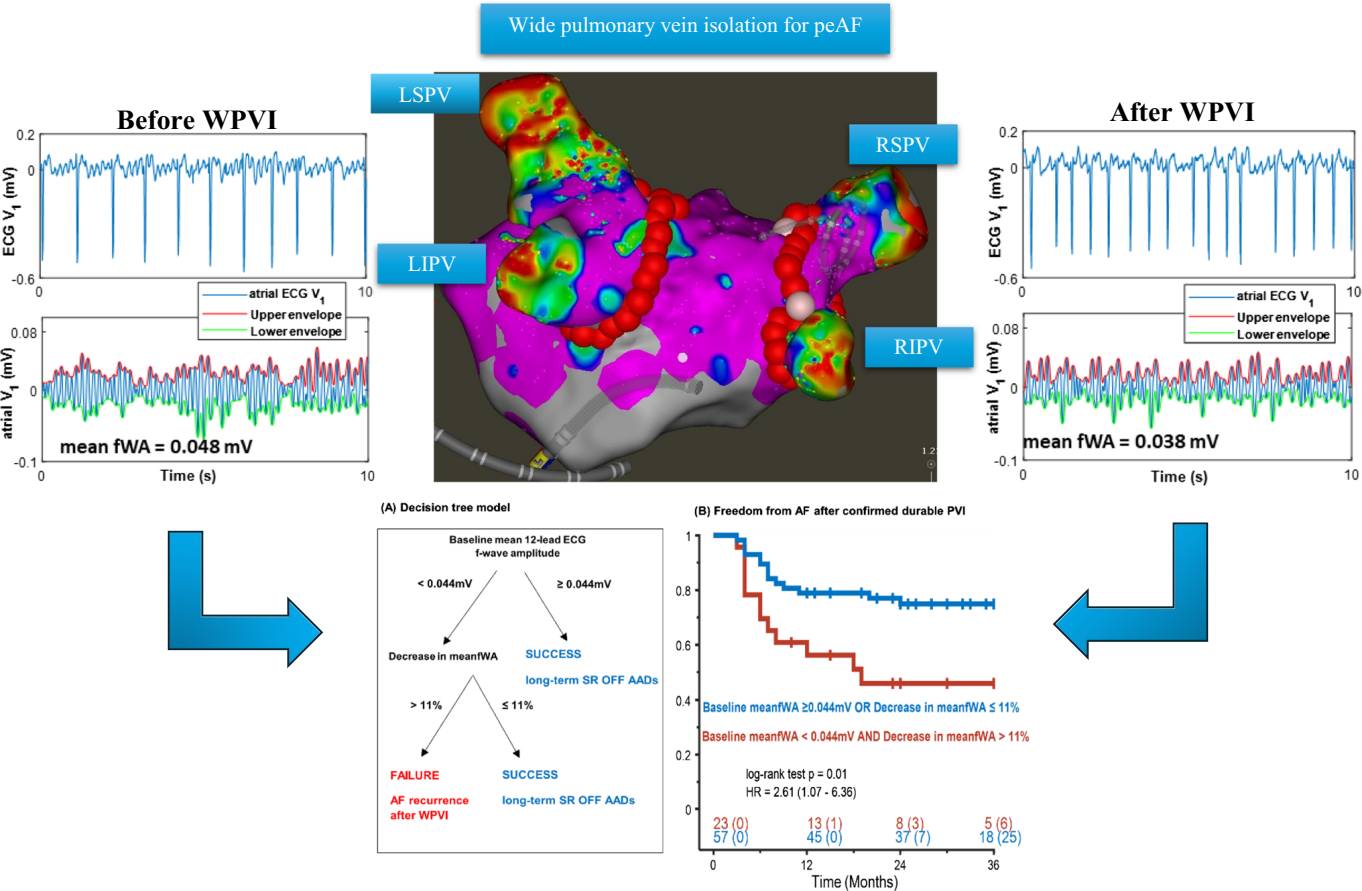

**Supplementary Information:**

The online version contains supplementary material available at 10.1007/s10840-025-02018-7.

## Introduction

Atrial fibrillation (AF) is the most common arrhythmia, significantly impacting quality of life and leading to serious comorbidities such as stroke and heart failure. Catheter ablation is the treatment of choice for paroxysmal AF [1], with successful abolition of AF in about 85% of the cases [2], after one or two procedures. However, in persistent AF (peAF), irreversible electroanatomical changes within the left atrium make the long-term success of catheter ablation more challenging [3]. Substrates beyond the pulmonary veins (PV) appear to sustain AF circuits in these patients. In patients with recurrent AF despite durable pulmonary vein isolation (PVI), neither substrate modification beyond PVs [4] nor advanced ablation strategies [5] at the redo procedure did convincingly improve arrhythmia-free survival. The challenge remains to select patients who would benefit most from durable wide-circumferential isolation of PVs (WPVI) to maintain sinus rhythm (SR) in the long term.

The atrial electrical activity during AF can be characterized using the amplitude, morphology, or frequency of the f-waves in the surface electrocardiogram (ECG). Various indices based on f-waves have been proposed as predictors of ablation outcome in peAF, but none demonstrated significantly better predictive power than that of the simple and intuitive amplitude of f-waves. The amplitude of f-waves (fWA) is unequivocally considered as being related to electro-anatomical factors, such as the amount of viable atrial myocytes and the underlying AF mechanism [6].

Herein, we first sought to evaluate the predictive accuracy of the baseline fWA for the long-term (> 24 months) maintenance of SR. Secondly, we studied how changes in fWA after WPVI reflect the underlying pathophysiological mechanism of AF maintenance and its relationship to long-term ablation outcomes.

## Methods

### Study population

This study has been performed within the framework of an ongoing project (REORGANIZE-AF) that is aimed at assessing the level of ECG organization in peAF to improve patients’ selection for WPVI [7, 8]. The study population consisted of 114 patients with continuous peAF > 1 month before ablation, referred for a first-time radiofrequency (RF) catheter ablation at three Swiss university hospitals. Patients were excluded if they were < 18 years old, pregnant, with severe valvular disease, or known infiltrative heart disease. All patients provided written informed consent before enrolment. The study protocol was approved by the local Human Research and Ethics Committees.

### Index ablation procedure and electrophysiological study

All patients underwent a de novo WPVI. The antiarrhythmic drugs (AADs) were discontinued for at least 5 half-lives before the procedure, except amiodarone (*n* = 26). The procedure was carried out under conscious sedation or general anesthesia. Electroanatomical mapping and 3D reconstruction of the left atrium (LA) were performed using the CARTO® 3 system (Biosense Webster®, Diamond Bar, CA). For each patient, the LA volume was computed after performing respiratory compensation and exclusion of the PVs [9]. A circumferential duodecapolar Lasso® catheter (electrode spacing 2–6-2 mm, Biosense Webster, Irwindale, CA) was used to map the PVs and the LA. Irrigated tip ablation catheters with contact force-sensing technology (Thermocool SmartTouch and SmartTouch SF®, Biosense Webster, Irwindale, CA) were used for ablation. Point-by-point RF applications in power control mode guided by minimum ablation index values were applied until PVI with a bidirectional block was achieved. Patients in whom peAF was not spontaneously converted into SR during ablation were electrically cardioverted. No extra-PV ablation was permitted, except for the ablation of the cavotricuspid isthmus in case of typical or reverse typical atrial flutter. After the index procedure, patients were followed up, and data were recorded using ≥ 48 h Holter-ECG at 3, 6, 12, 18, and 24 months, then every year. Recurrence was defined as AF or atrial tachycardia (AT) lasting more than 30 s [2].

### Redo-ablation

A second ablation was performed in patients with AF or AT recurrence after a 3-month blanking period. A multipolar catheter (Lasso or PentaRay catheter, Biosense Webster®) was used to verify successful PV isolation. For reconnected PVs, RF ablation (Thermocool SmartTouch or QDOT, Biosense Webster®) was performed at the reconnection site to ensure durable PVI and bi-directional conduction block. For patients without PV reconnections, the decision regarding the further ablation strategy was left to the discretion of the operator. After redo ablation, the patients were monitored for up to 24 months.

### Primary clinical endpoint and patient groups

The primary clinical endpoint was long-term freedom from AF OFF AADs after WPVI. The study population was divided into two groups:

- “SUCCESS” group consisted of patients with long-term (> 24 months) maintenance of SR without AADs after one or two PVI procedures.
- “FAILURE” group consisted of patients with recurrence of AF or AT despite confirmed durable PVI at the redo procedure.

### ECG processing

**Fig. 1:**
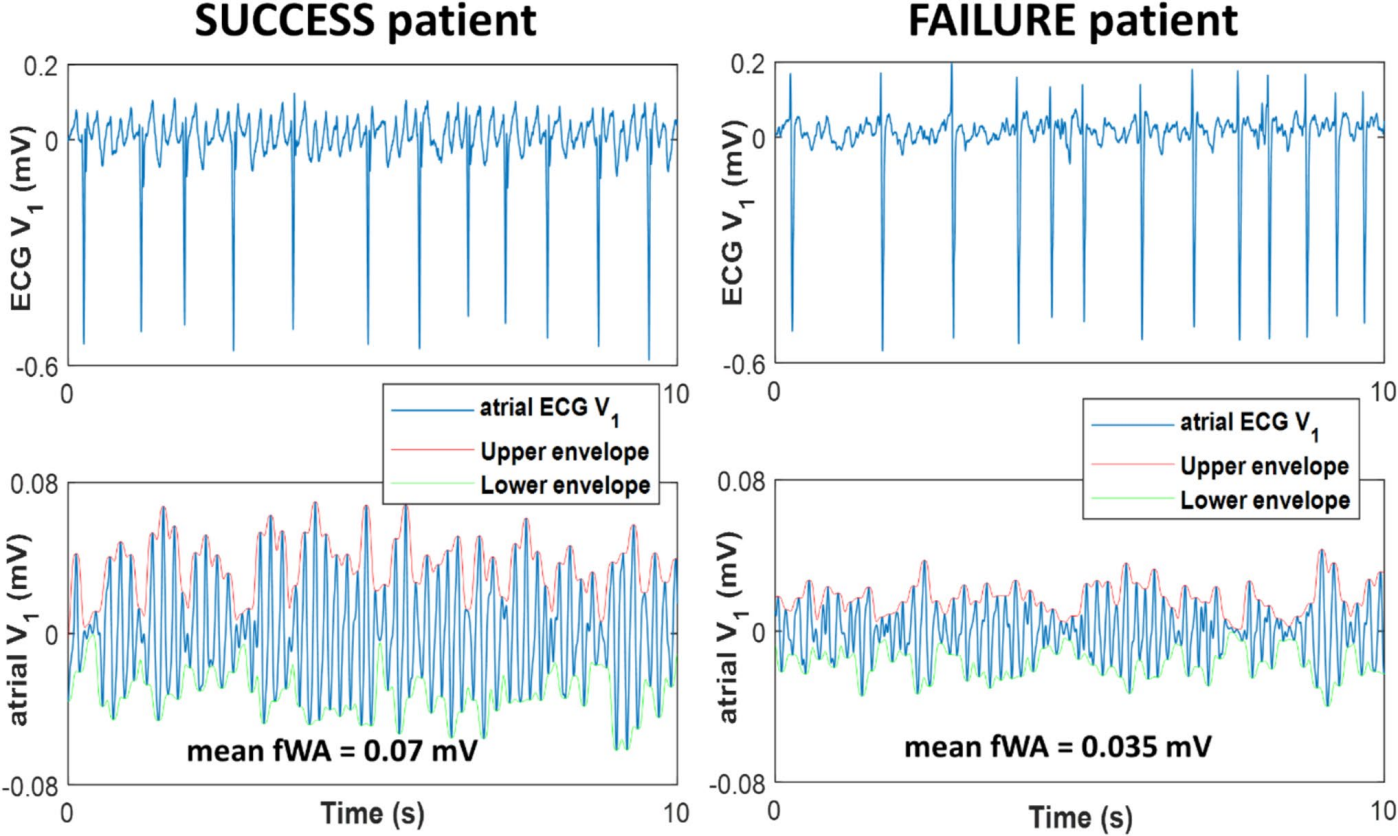
Estimation of the amplitude of f-waves (fWA) on 10-s ECG lead V_1_ in a SUCCESS patient (left) and a FAILURE patient (right). Upper panels represent surface ECG lead *V*_1_ after baseline wander correction. Lower panels illustrate ECG lead *V*_1_ after QRST cancellation (atrial signal). Mean fWA was computed as the average difference between the upper (red) and lower (green) envelopes of the f-wave signal. mV, milivolt

Standard 12-lead ECG was continuously monitored during the ablation procedures using the Axiom Sensis XP® System (Siemens®, Munich, Germany) at a sample rate of 2 kHz and bandpass filter settings of 0.5–200 Hz. The ECG chest lead *V*_6_ was placed on the back (*V*_6b_) of the patients, within the cardiac silhouette, to improve LA activity recording [10]. One-minute ECG signals recorded before the index procedure (baseline) and 1-min ECG signals recorded at the end of the index procedure (before cardioversion or conversion of AF into SR) were retrospectively processed using MATLAB® (The Mathworks Inc., Natick, MA, USA). The ECG signals were bandpass-filtered (1–50 Hz) to remove baseline wander and power line interference. The atrial ECG signals were obtained by removing the QRST waves from the surface ECG using the single beat method in which the QRS and T waves are treated separately [11]. The local maxima and minima of the f-wave signal on the 100-ms sliding window were detected on each ECG lead signal. The upper and lower envelope of the atrial ECG signal was estimated by lowpass filtering each time series of local extrema. The temporal average of f-wave amplitude (fWA [mV]) on each ECG lead was computed as the average difference between the upper and lower envelope. Figure 1 shows illustrative examples of fWA estimation on 10-s epochs of atrial ECG signal recorded on the ECG lead *V*_1_ from a SUCCESS patient (left panel) and a FAILURE patient (right panel), respectively.

### Statistical analysis

Continuous variables were expressed as the median and interquartile range (IQR) and categorical variables as numbers and percentages. The significance of any difference between groups was analyzed with the Mann–Whitney *U*-test for continuous variables and with Pearson’s chi-squared test or Fisher’s exact test for categorical variables. A receiver-operator characteristic (ROC) analysis was performed to assess the performance of fWA as a predictor of ablation outcome. The optimal ROC curve cutoff was defined as the combination of the highest sensitivity and specificity. A logistic regression analysis was used to determine the predictors of ablation outcome and to compute the respective odds ratios (OR). Freedom from atrial arrhythmias during follow-up was analyzed using the Kaplan–Meier method, and a log-rank test was applied to compare differences between groups. A decision tree model based on baseline mean fWA and its relative changes following WPVI was developed to intra-procedurally predict ablation outcomes. The statistical significance was set at *p* < 0.05. Analyses were performed in MATLAB® (The Mathworks Inc., Natick, MA, USA).

## Results

### Study population

**Fig. 2:**
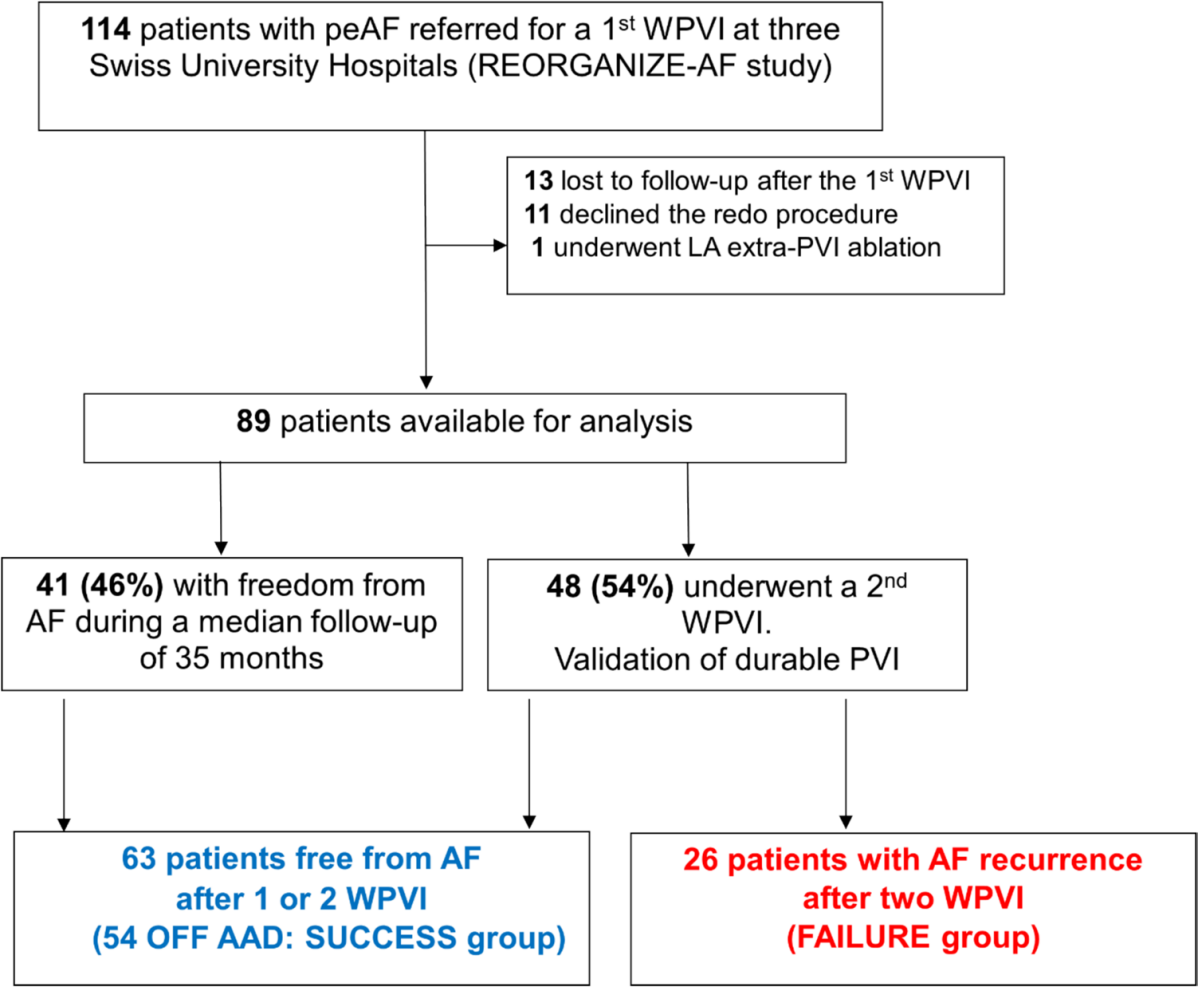
Study population overview. peAF, persistent atrial fibrillation; FU, follow-up; LA, left atrium; WPVI, wide pulmonary vein isolation

Of 114 patients included in the REORGANIZE-AF study who underwent a de-novo WPVI, one patient was excluded because of extra-PV LA ablation at the index procedure, 13 patients were lost to follow-up, and 11 declined the redo ablation. Accordingly, 89 patients remained for analysis (Fig. 2). They suffered from AF for 3 ± 4 years, with peAF for 7 ± 7 months (18% long-standing persistent AF and 82% persistent AF; Supplementary Table S1).

Over a mean follow-up of 35 ± 10 months, freedom from AF OFF AADs was achieved in 61% (54/89) of patients (SUCCESS group; 38 after a single WPVI and 16 patients after redo WPVI). Ten percent (9/89) of patients were free from AF ON AAD after 1 or 2 WPVI, while 29% (26/89) of patients had AF recurrence after the redo procedure (FAILURE group). The study overview is presented in Fig. 2. Table 1 and Supplementary Table S1 report the clinical characteristics of the subgroups. No significant differences were observed between the FAILURE and SUCCESS groups, except for AAD use during follow-up and diabetes (Table 1). After including patients who maintained SR ON AADs during FU, patients with AF recurrence after redo WPVI had longer duration in sustained AF and received AADs more frequently during follow-up compared to patients in SR ON and OFF AADs after one or two WPVI procedures (duration in sustained AF: 10 (3, 23) months vs. 6 (2, 24) months, *p* < 0.05; AADs at FU: 42% vs. 14%, *p* < 0.05; Supplementary Table S1).

**Table 1:** Clinical characteristics of the SUCCESS and FAILURE groups

Characteristics	Overall, *N* = 80^1^	SUCCESS (*N* = 54)^1^ AF freedom OFF AADs	FAILURE (*N* = 26)^1^ AF recurrence after redo WPVI	*p*-value^2^
**Age**	64 (46, 74)	64 (44, 75)	63 (48, 73)	0.56
**Follow-up duration (mo)**	36 (16, 53)	36 (23, 51)	34 (13, 52)	0.59
**AADs at FU (%)**	11 (14)	0 (0)	11 (42)	< 0.001
**Duration in sustained AF (mo)**	7 (2, 24)	6 (2, 24)	10 (3, 23)	0.074
**Type of AF**				
➢ **Long-standing persistent (%)**	14 (17)	9 (17)	5 (19)	> 0.99
➢ **Persistent (%)**	66 (83)	45 (83)	21 (81)	
**Time since AF diagnosis (yrs)**	3.0 (1.0, 12.1)	3.0 (1.0, 12.4)	3.0 (1.0, 11.3)	0.75
**Female/male (%)**	18/62 (23, 78)	9/45 (17/83)	9/17 (35, 65)	0.072
**LVEF (Simpson method, in %)**	55 (25, 65)	55 (25, 65)	55 (25, 65)	0.67
**Left atrial volume (ml)**	119 (71, 193)	117 (73, 165)	122 (73, 196)	0.53
**LAVI (mL/m**^**2**^**)**	57 (35, 90)	57 (35, 85)	55 (35, 93)	0.66
**BMI (kg/m**^**2**^**)**	28.0 (21.0, 39.2)	28.5 (21.0, 35.4)	28.0 (23.0, 42.8)	0.27
**Hypertension**	5 (6.3)	5 (9.3)	0 (0)	0.17
**CAD (%)**	8 (10)	7 (13)	1 (3.8)	0.26
**HCM (%)**	2 (2.5)	2 (3.7)	0 (0)	> 0.99
**VHD (%)**	8 (10)	5 (9.3)	3 (12)	0.71
**OSAS (%)**	35 (44)	25 (46)	10 (38)	0.67
**Diabetes (%)**	9 (11)	9 (17)	0 (0)	0.027
**Tobacco (%)**	19 (24)	12 (22)	7 (27)	0.64
**Hypercholesterolemia (%)**	27 (34)	21 (39)	6 (23)	0.21
**Medication at baseline (index PVI)**				
➢ **Betablocker (%)**	61 (76)	43 (80)	18 (69)	0.31
➢ **AADs (%)**	25 (32)	16 (31)	9 (36)	0.65
➢ **CCB (%)**	7 (8.8)	3 (5.6)	4 (15)	0.21
➢ **ACE inhibitor (%)**	22 (28)	15 (28)	7 (27)	0.94
➢ **Statin (%)**	11 (14)	9 (17)	2 (7.7)	0.49
➢ **Angiotensin receptor antagonist (%)**	9 (11)	6 (11)	3 (12)	> 0.99
**Cumulative ablation time of index procedure (sec)**	2091 (1251–3534)	2,111 (1249–3461)	2071 (1288–4139)	0.74

^1^Median and IQR (5–95%) or frequency (%)

*AADs* antiarrhythmics, *ACE* angiotensin-converting enzyme, *PVI* pulmonary vein isolation, *FU* follow-up, *LAVI* left atrial volume index, *CAD* coronary artery disease, *HCM* hypertrophic cardiomyopathy, *VHD* valvular heart disease, *OSAS* obstructive sleep apnea syndrome, *CCB* calcium channel blocker

^2^Mann–Whitney *U*-test; Pearson’s chi-squared test; Fisher’s exact test

### Effect of WPVI on f-wave amplitude

**Fig. 3:**
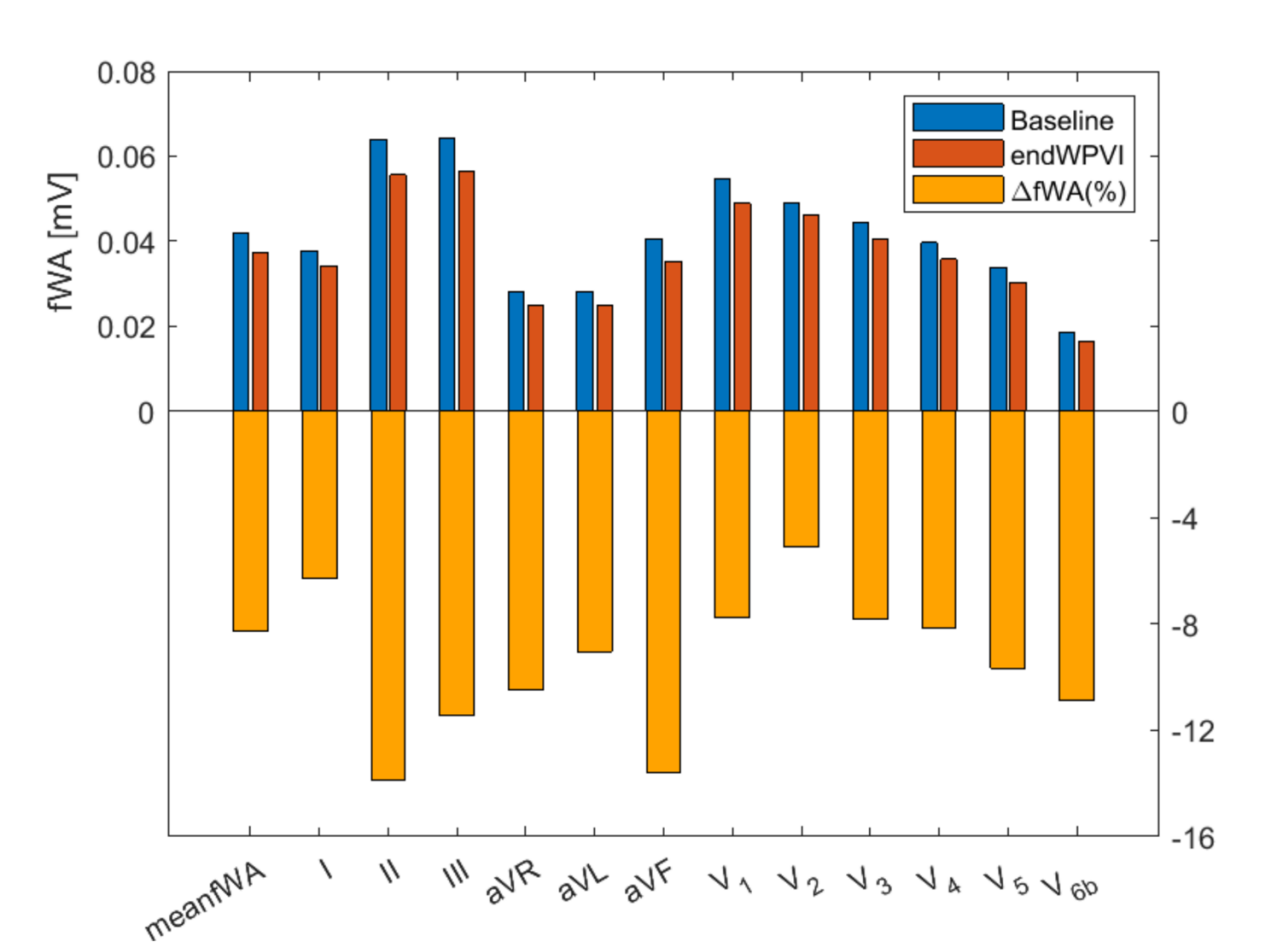
Effect of WPVI on f-wave amplitude on the study cohort (*n* = 89). Mean fWA across the 12 ECG leads (meanfWA) and individual ECG lead fWA values at baseline (blue color) and at endWPVI (red color). Relative change (ΔfWA%) in fWA at endWPVI from the baseline fWA value is indicated in orange color

Figure 3 shows fWA at baseline and at endWPVI for the cohort of 89 patients. The fWA values are presented for each individual ECG lead, along with the mean (meanfWA) across the 12-lead ECG and the relative change (%) in fWA following WPVI (calculated as the percentage deviation of the fWA at endWPVI from the baseline fWA values).

The baseline meanfWA across the 12-lead ECG was 0.041 ± 0.011 mV, with the smallest value on lead *V*_6b_ (0.018 ± 0.006 mV) and the highest one on lead III (0.064 ± 0.020 mV). At endWPVI, the meanfWA was 0.037 ± 0.011 mV, and the individual lead fWA values ranged from 0.016 ± 0.006 mV on lead *V*_6b_ to 0.056 ± 0.02 mV on lead III. A decrease in fWA following WPVI was observed on all ECG leads. The highest decrease in fWA was observed on lead II (relative change of − 14 ± 22%), and the smallest one on lead *V*_2_ (relative change of − 5 ± 19%). Importantly, similar results were also obtained on the entire group of 114 patients undergoing a first-time WPVI for peAF (Supplementary Figure S1).

### Impact of f-wave amplitude on long-term ablation outcome

Table 2 shows the ECG leads displaying significant differences in fWA between the SUCCESS and FAILURE groups. fWA values for all ECG leads are shown in Supplementary Tables S2 and S3. The SUCCESS group showed significantly higher fWA values in ECG lead *V*_1_, *V*_4_, and *V*_5_ at baseline, and in ECG lead III, aVL, aVF, and *V*_4_ at endWPVI than those of the FAILURE group (*p* < 0.05). Moreover, the FAILURE group displayed a significantly lower meanfWA across the 12-lead ECG (*p* = 0.018) at endWPVI and a greater reduction in fWA following WPVI in the ECG lead III and aVL than those of the SUCCESS group (*p* = 0.036 and 0.023, respectively).

**Table 2:** ECG leads to significant differences in f-wave amplitude between groups

		SUCCESS (freedom from AF OFF AAD) *N* = 54	FAILURE (AF recurrence after redo WPVI) *N* = 26	*p*-value
**Before WPVI (baseline)**	V_1_	0.050 (0.044, 0.061)	0.043 (0.035, 0.054)	0.045
	V_4_	0.040 (0.033, 0.047)	0.035 (0.029, 0.039)	0.023
	V_5_	0.034 (0.028, 0.040)	0.028(0.024, 0.033)	0.023
**After WPVI (endWPVI)**	III	0.057 (0.045, 0.072)	0.044 (0.036, 0.054)	0.004
	aVL	0.025 (0.020, 0.032)	0.020 (0.017, 0.024)	0.006
	aVF	0.032 (0.027, 0.045)	0.029 (0.022, 0.034)	0.025
	V_4_	0.035 (0.029, 0.045)	0.030 (0.023, 0.038)	0.038
	meanfWA	0.038 (0.031, 0.046)	0.032 (0.027, 0.037)	0.018
**Relative changes (%) after WPVI in fWA after WPVI**	aVL	− 6% (− 16, 1)	− 16% (− 31, − 3)	0.023
	III	− 8% (− 18, 1)	− 17% (− 25, − 3)	0.036

Values are median (25th and 75th percentiles) in mV. meanfWA denotes the mean of fWA values across the 12 ECG leads. Other abbreviations as in the previous figures and tables

### f-wave amplitude as a predictor of long-term ablation outcome

Table 3 reports the predictive performances of fWA at baseline and endWPVI for long-term ablation outcomes. Univariate logistic regression analysis showed that high fWA values in lead *V*_4_ (OR 1.72; *p* < 0.05) at baseline, and in lead III, aVL, and aVF (OR 1.52, 2.77 and 1.69; *p* < 0.05) at endWPVI were significantly associated with long-term freedom from AF OFF AADs. Moreover, high mean fWA of the 12-lead ECG at endWPVI and a decrease in fWA following WPVI of < 2.6% in lead aVL or < 3.3% in lead III were also significantly associated with long-term AF freedom after durable PVI (*p* < 0.05).

**Table 3:** f-wave amplitude at baseline and following WPVI as a predictor of long-term ablation outcome

		Odds ratio		ROC analysis					
		OR (95% CI)	*p*-value	AUC (95%CI)	Optimal cut-off [mV]	SE	SP	PPV	NPV
**Before WPVI (baseline)**	V_1_	1.18 (0.92, 1.52)	0.17	64 (55; 72)	≥ 0.053	74	57	75	38
	V_4_	1.72 (1.04, 2.84)	0.03	66 (57; 74)	≥ 0.036	51	80	74	44
	V_5_	1.56 (0.91, 2.64)	0.10	65 (56; 73)	≥ 0.030	57	76	79	44
**After WPVI (endWPVI)**	III	1.52 (1.1; 2.12)	0.01	70 (61, 78)	≥ 0.046	50	84	78	48
	aVL	2.77 (1.28; 6.09)	0.01	69 (60; 77)	≥ 0.024	70	72	86	45
	aVF	1.69 (1.01; 2.54)	0.05	66 (57; 74)	≥ 0.042	90	42	86	38
	V_4_	1.52 (0.93; 2.48)	0.09	65 (56; 73)	≥ 0.034	64	68	77	42
	meanfWA	1.76 (1.03; 3.03)	0.03	67 (58; 73)	≥ 0.031	41	92	71	55
**Relative changes (%) after WPVI**	aVL	1.04 (1.01; 1.07)	0.02	66 (54; 74)	≤ 2.6%	74	52	82	40
	III	1.04 (1.01; 1.07)	0.03	66 (56; 73)	≤ 3.3%	76	52	80	38

*AUC* area under the curve, *OR* odds ratio, *ROC* receiver operating characteristic, *SE* sensitivity, *SP* specificity, *PPV* positive predictive value, *NPV* negative predictive value. Other abbreviations as in the previous figures and tables

### A decision tree model based on 12-lead ECG mean fWA for predicting ablation outcomes

**Fig. 4:**
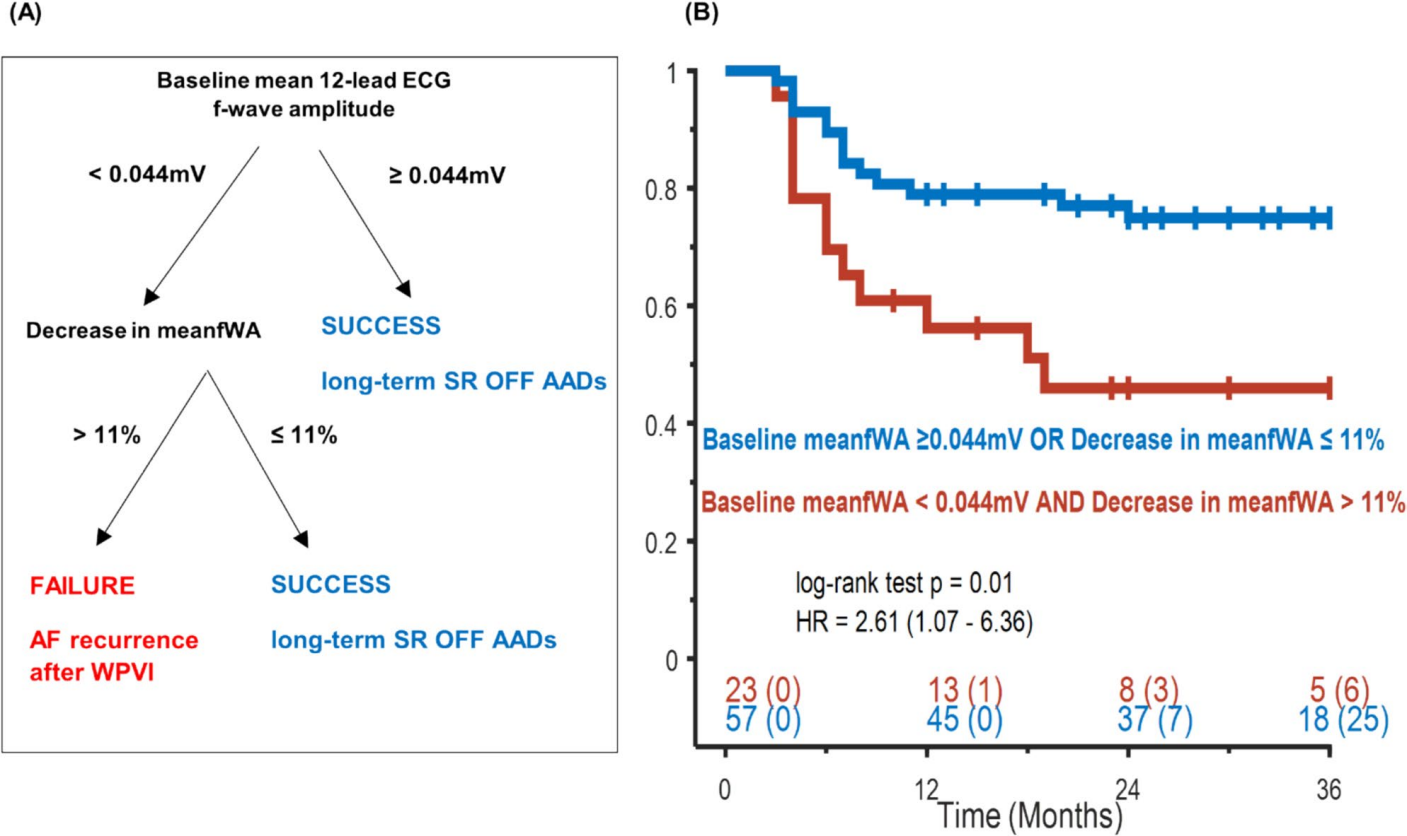
Predictive performance of 12-lead ECG meanfWA for long-term SR maintenance. **A** Decision tree model based on baseline meanfWA and its relative changes following WPVI. **B** Kaplan–Meier curves for freedom from AF recurrence after confirmed durable PVI. Numbers at the bottom indicate patients at risk and censored, respectively. Abbreviations as in previous figures

Table 3 indicates that no ECG lead serves as a significant predictor of long-term ablation outcomes at both baseline and after WPVI. Hence, a decision-tree model was created (Fig. 4A) to incorporate both baseline 12-lead ECG meanfWA and its corresponding relative change following WPVI, aiming to encompass the entire atrial ECG f-wave information. This model was based on two steps: (1) a baseline meanfWA ≥ 0.044 mV or a decrease in meanfWA ≤ 11% following WPVI-identified patients with a high likelihood of maintaining long-term SR after durable PVI, with a sensitivity of 81%, a specificity of 63%, a PPV of 75%, and a NPV of 54%; (2) patients with a baseline meanfWA < 0.044 mV and, simultaneously, a decrease in meanfWA > 11% had significantly higher AF recurrence rate compared to other patients (30% vs. 56% in SR, *p* = 0.01, Fig. 4B). In summary, low baseline fWA values and a strong decrease in fWA following WPVI are associated with unfavorable long-term ablation outcomes.

## Discussion

### Main findings

This study yields important new findings regarding the clinical role of the surface ECG f-wave amplitude in predicting long-term outcomes after WPVI in peAF. First, we confirm that low fWA is associated with unfavorable long-term ablation outcomes. Second, fWA following WPVI appears more appropriate than the baseline fWA to identify patients at high risk of AF recurrence at follow-up. Importantly, patients with high baseline fWA and a small decrease following WPVI are more likely to remain in SR after confirmed durable PVI. Third, a decision tree based on meanfWA measured on baseline 12-lead ECG and its changes following WPVI showed good performance in identifying patients in whom WPVI alone is sufficient to maintain long-term SR. Altogether, these findings suggest that measuring fWA before and after WPVI may help characterize the underlying AF substrate. This may help to intra-procedurally identify patients with peAF who are unresponsive to WPVI alone and may benefit from additional substrate ablation beyond PVI.

### Relationship between baseline f-wave amplitude and long-term ablation outcome

High baseline fWA values are linked to a high rate of acute AF termination or improved ablation outcome [12]. However, there is a certain heterogeneity between the previous findings, as no single ECG lead consistently demonstrated superiority over others in predicting ablation outcomes. This inconsistency is primarily due to the high spatial variability of f-wave content across different ECG leads. Park et al. identified an fWA cutoff value of < 0.061 mV in *V*_1_ as a predictor for AF recurrence after PVI and ablation of complex fragmented atrial electrograms in patients with non-paroxysmal AF [13], whereas Nault et al. observed higher AF recurrence rate after stepwise ablation when fWA < 0.05 mV in lead *V*_1_ [6]. Zarsoso et al. showed that a combination of multiple leads (I, *V*_1_, *V*_2_, and *V*_5_) improved the prediction of long-term ablation outcomes as compared to single-lead-based prediction [14]. In our study, the baseline mean fWA across the 12-lead ECG was not significantly associated with WPVI outcome. Nevertheless, we found that patients free from AF at follow-up displayed higher baseline fWA values on the ECG leads *V*_1_, *V*_4_, and *V*_5_ than those of patients with recurrence. The logistic regression analysis identified only a single lead, *V*_4_, significantly associated with long-term maintenance of SR after WPVI. Altogether, these results support the hypothesis that surface ECG fWA carries valuable clinical information and may serve as a predictor of ablation outcomes. However, the high heterogeneity among the findings highlights the challenges of integrating fWA measurement into clinical practice.

### Effect of WPVI on f-wave amplitude

A common belief is that the reduction in fWA following ablation is primarily linked to the decrease in viable atrial tissue, which is supported by studies reporting a decrease in both P-wave amplitude and duration after ablation [15]. Based on the solid angle theory [16], any ECG lead represents a mixture of both electrical atrial activities; hence, ECG leads should respond differently to local ablation lesions. Lankveld et al. found a significant decrease in fWA in lead *V*_6_ following PVI, while the fWA in lead *V*_1_ slightly increased and then decreased during subsequent RA ablation [12]. In contrast, Squara et al. did not find significant changes in fWA after stepwise ablation [17]. In our study, WPVI led to a decrease in the fWA across all ECG leads. However, the relative changes in fWA amplitude differed between groups. FAILURE patients exhibited a significant reduction in fWA following WPVI, whereas SUCCESS patients showed little or absent fWA reduction. This divergent response to PVI led to more notable differences in fWA between groups at the end of WPVI than those observed at baseline. After WPVI, the SUCCESS group exhibited significantly higher fWA values compared to the FAILURE group across multiple ECG leads (III, aVL, aVF, and V4) as well as in the meanfWA across the entire 12-lead ECG. The degree of fWA reduction in ECG leads III and aVL following WPVI was an independent predictor of AF recurrence during follow-up. Importantly, only ECG lead *V*_4_ showed a significant difference in f-wave amplitude between groups, both at baseline and after WPVI. The reasons why these specific ECG leads showed predictive value remain unclear, as they do not specifically reflect LA or RA electrical activity [9]. Moreover, these results align with studies indicating that LA regions with significant interstitial fibrosis, resulting from advanced atrial cardiomyopathy, are more extensive in patients unresponsive to WPVI alone compared to patients without recurrence. Atrial fibrosis, characterized by loss of myofibrillar cells and increased intracellular space, was significantly associated with LA voltage reduction, as shown in a recent study by Takahashi and colleagues [18]. Fibrosis and increased intracellular space were also associated with long-standing persistent compared with paroxysmal AF, as well as higher rate of atrial tachyarrhythmia recurrences after ablation. Well-established clinical risk factor such as left atrial volume index (LAVI) was also significantly associated with low voltage area within the LA [18]. In our study, a reduced fWA after WPVI appears to reflect the abnormal electrical activity of the remaining remodelled atrial tissue, which may play a role in AF recurrence despite durable WPVI. As such, measurements of fWA after PVI may offer valuable insights into the degree of electrical remodelling in the atrial tissue that contributes to AF maintenance.

### Changes in f-wave amplitude following PVI and long-term ablation outcome

We found that a decision-tree model combining the baseline mean fWA across the 12-lead ECG and its reduction in amplitude following WPVI provides a better group separation than that obtained with a single ECG lead. Among patients with a low baseline meanfWA < 0.044 mV, those with a decrease in meanFWA > 11% had the highest risk of AF recurrence. While low baseline fWA may fit with extensive bi-atrial electrical remodelling, further fWA reduction following PVI appears to reflect the advanced electrical remodelling of the remaining atrial tissue outside the PVs [18, 19]. The surface ECG fWA depends on the magnitude of the underlying atrial voltage. This voltage, in turn, is influenced by the extent of viable atrial muscle and the frequency of re-entrant waves during AF [20]. Lessons from the DECAAF II RCT showed that the extent and repartition of atrial fibrosis, its histological subtypes, and arrhythmogenic properties can be very heterogenous in persistent AF [21]. This could explain why the reduction in fWA was more pronounced in the FAILURE group following WPVI. Both extra-PVs foci and specific LA regions exhibiting significant fibrosis due to advanced atrial cardiomyopathy may be the primary contributors to the residual surface f-waves, thereby leading to a greater decrease in fWA after WPVI.

In summary, a decision-tree model that integrates baseline fWA and its relative changes post-PVI has the potential to intra-procedurally identify patients who could benefit from extra-PV ablation to improve long-term SR maintenance.

### Relationship between f-wave amplitude and other clinical predictive markers of ablation outcome

Increased LA volume or LAVI has been independently associated with less favorable ablation outcomes [22, 23]. In our study, LA volume and LAVI were comparable between patients maintaining long-term SR ON AADs after WPVI and those experiencing AF recurrence following redo WPVI. Additionally, no statistically significant relationship was observed between fWA and either LA volume or LAVI (Supplementary Figure S2). Several factors may account for these divergent and heterogeneous findings. The median LAVI in both the SUCCESS and FAILURE groups indicated severe LA dilatation (> 48 mL/m^2^), a characteristic commonly observed in populations with persistent or long-standing persistent AF. LAVI reflects structural remodelling of the LA, increasing with prolonged AF duration and influenced by clinical markers of AF recurrence and comorbidities, including obesity, hypertension, diabetes, and mitral valve disease [24–27]. Conversely, the amplitude of surface ECG f-waves may reflect structural remodelling, such as fibrosis, or electrical remodelling driven by residual AF triggers post-PVI. This supports the notion that LAVI and fWA are independent markers of AF recurrence. Indeed, a meta-analysis of observational studies [23] demonstrated that while AF recurrence is more frequent in patients with elevated LAVI, the attributable risk is modest, suggesting LAVI may not be a robust clinical marker for patients’ selection for catheter ablation. Recent data [28] indicated that patients with persistent AF and mild LA dilatation (LAVI > 34 mL/m^2^) achieved similar 1-year outcomes to those with paroxysmal AF and moderate/severe LA dilatation.

Prior studies have also identified AF duration as an independent predictor of PVI failure, particularly in patients with persistent or long-standing persistent [29]. However, in our cohort, neither sustained AF duration nor AF type (persistent vs. long-standing persistent) was associated with long-term ablation outcomes or correlated with fWA. These results may be attributed to the fact that AF can be asymptomatic, and therefore, the AF onset or the duration in sustained AF cannot be reliably established in clinical practice.

Altogether, these findings support the idea that, due to the complex nature of AF, the predictive performance of individual clinical or ECG-based markers is significantly influenced by patient-related factors, such as population heterogeneity, follow-up duration, and ablation endpoints [30].

### Clinical implications

Surface fWA is intuitive and straightforward to calculate from ECG leads, making it practical for integration into clinical settings. However, the pathophysiological mechanisms underlying the generation of surface f-waves remain unclear. Some studies have suggested that fWA may reflect atrial tissue viability [16], and others positioned it as part of the AF “complexity markers” [12]. Despite their moderate predictive accuracy, parameters such as maximum or mean fWA have been linked to ablation outcomes [6]. Our study proposes a composite approach that integrates baseline mean fWA across all ECG leads with its relative changes post-ablation to improve the prediction of long-term outcomes following WPVI in persistent AF. While this approach may not be suitable for pre-ablation patient selection, it holds significant potential for enhancing intra-procedural decision-making regarding the need for redo ablation following AF recurrence. By identifying patients who may benefit from additional ablation beyond PVI—such as posterior wall isolation, particularly with emerging pulse-field ablation (PFA) technologies—this novel model addresses a critical need for procedural personalization. Prospective validation in an external cohort is required to confirm the efficacy of our decision model and its utility in guiding substrate ablation beyond PVI.

## Limitations

First, this study is limited by the small size of the population, which might result in overestimated performances of the predictive models. Nonetheless, the multicentric nature of our study helped us reduce possible selection bias. Furthermore, fWA was calculated automatically, eliminating any potential bias stemming from manual measurements. Second, we did not compare surface fWA to other metrics of AF complexity or to the extent of endocardial low-voltage areas. There is evidence that the reduction in amplitude of atrial ECG signal correlates with the amount of atrial low voltage area [17, 31], fibrosis, and advanced atrial remodelling [18]. Our primary objective was to assess the clinical utility of the fWA in identifying patients in whom WPVI alone suffices to restore long-term SR, without focusing on deciphering the pathophysiological mechanisms underlying persistent AF. However, the current study revealed no statistically significant relationship between fWA and LA volume, LAVI, or duration in sustained AF (Supplementary Figure S2), which is consistent with the findings of Ishihara et al. [32]. Third, our cohort included only patients who underwent point-by-point radiofrequency WPVI. Further studies are needed to confirm the clinical predictive value of fWA for emerging ablation techniques for AF, such as PFA.

## Conclusion

This study demonstrates that a high amplitude of surface ECG f-waves, both before and after WPVI, is associated with a high success rate of long-term SR maintenance (> 24 months after WPVI). Conversely, patients with low baseline f-wave amplitude and a significant reduction in fWA post-WPVI exhibit a high rate of AF recurrence. This suggests advanced atrial remodelling, indicating that additional substrate modifications beyond the PVs may be necessary to achieve long-term sinus rhythm restoration.

### Clinical perspectives

This study highlights the significant potential of fWA on 12-lead ECG as a predictor of long-term ablation outcomes in patients with peAF. Our composite approach—integrating baseline fWA with its dynamic changes—could improve the prediction of ablation success and inform personalized treatment strategies beyond PVI. Incorporating this noninvasive tool into routine clinical practice could enhance the precision of patient selection for ablation, ultimately leading to more tailored and effective ablation strategies in persistent AF.

## Supplementary Information

Below is the link to the electronic supplementary material.

Supplementary file1 (DOCX 595 KB)

## Data Availability

The data underlying this article cannot be shared publicly due to ethical reasons. The data will be shared on request to the corresponding author with permission of Lausanne University Hospital.

## Abbreviations

AAD: Antiarrhythmic drug
AF: Atrial fibrillation
peAF: Persistent atrial fibrillation
AFL: Atrial flutter
AT: Atrial tachycardia
ECG: Electrocardiogram
fWA: F-wave amplitude
IQR: Interquartile range
LA: Left atrial/atrium
LAVI: Left atrial volume index
LVEF: Left ventricular ejection fraction
NPV: Negative predictive value
OR: Odds ratios
PPV: Positive predictive value
PV: Pulmonary vein
PVI: Pulmonary vein isolation
RFA: Radiofrequency ablation
ROC: Receiver-operator characteristic
SR: Sinus rhythm
WPVI: Wide circumferential isolation of pulmonary veins
