# Supplementary material for: Predicting outcomes in persistent atrial fibrillation: the impact of surface ECG f-wave amplitude following pulmonary vein isolation
Source: J Interv Card Electrophysiol. 2025 Feb 19;68(6):1243–55. doi: 10.1007/s10840-025-02018-7 (PMC12399694; doi:10.1007/s10840-025-02018-7)
Supplement: Supplementary file 1 — Supplementary file1 (DOCX 595 KB) [file 10840_2025_2018_MOESM1_ESM.docx]

**Supplementary Results**

**Title: Predicting Outcomes in Persistent Atrial Fibrillation: The Impact of Surface ECG F-Wave Amplitude Following Pulmonary Vein Isolation**

**Journal of Interventional Cardiac Electrophysiology**

**Aruran Baskaralingam, MD^a^, Matteo Marchetti, MD^a^, Jorge Solana-Munoz, MSc^a^, Cheryl Teres, MD^a^, Mathieu Le Bloa, MD^a^, Alessandra Pia Porretta, MD^a,b^, Giulia Domenichini, MD^a^, Ciro Ascione, MD^a^, Laurent Roten, MD^c^, Sven Knecht, PhD^4^,** **Michael Kühne, MD^d^, Christian Sticherling, MD^d^, Patrizio Pascale, MD^a^, Etienne Pruvot, MD^a*^, Adrian Luca, PhD^a*^**

^a^ Service of Cardiology, Lausanne University Hospital and University of Lausanne, Lausanne, Switzerland

^b^ Service of Cardiology, APHP Hôpital Bichat, Paris, France

^c^ Department of Cardiology, Inselspital, Bern University Hospital, University of Bern, Bern, Switzerland

^d^ Department of Cardiology, University Hospital of Basel, Basel, Switzerland

* Shared senior authorship

**Running title:** ECG F-Wave Amplitude to Predict Outcomes Post-PVI in Persistent AF

**Address for correspondence**

**Aruran Baskaralingam**

[**aruran006@hotmail.com**](mailto:aruran006@hotmail.com)

**Service de Cardiologie, Centre hospitalier universitaire vaudois (CHUV)**

**Rue du Bugnon 46, CH-1011 Lausanne, Vaud, Suisse**

**Phone: +41794439030**

## Clinical characteristics

- Supplemental Table S1 shows the baseline clinical characteristics of the study population according to the subgroups: AF freedom ON and OFF AADs vs AF recurrence after redo WPVI procedures.

## Amplitude of F-waves at baseline and at the end of WPVI

- Supplemental Figure S1 shows fWA values at baseline and at the end of WPVI for the entire population of 114 patients who underwent a first-time WPVI. A decrease in fWA following WPVI was observed on all ECG leads and in the mean fWA across the 12-lead ECGs as well.
- Supplemental Table S2 and S3 include comparisons between fWA values at baseline and at the end of WPVI, respectively, according to the primary clinical endpoint (SUCCESS vs FAILURE group).

**Relationship between fWA and other clinical predictive markers of ablation outcome**

- Supplemental Figure S2 shows the relationship between fWA and the duration in sustained AF, left atrium volume and left atrium volume index.

**Supplementary Table S1**  **Clinical characteristics of study population**

| **Characteristic** | **Overall**, N = 89^1^ | **AF freedom ON and OFF AADs**  **(N = 63)**^1^ | **AF recurrence after redo WPVI**  **(N = 26)**^1^ | **p-value**^2^ |  |
| --- | --- | --- | --- | --- | --- |
| **Age** | 64 (46, 74) | 65 (45, 74) | 63 (48, 73) | 0.37 |  |
| **Follow-up duration (mo)** | 35 (16, 52) | 35 (3, 50) | 34 (13, 52) | 0.70 |  |
| **AADs at FU (%)** | 20 (22) | 9 (14) | 11 (42) | 0.004 |  |
| **Duration of sustained AF (mo)** | 7 (2, 24) | 6 (2, 24) | 10 (3, 23) | 0.047 |  |
| **Type of AF** |  |  |  |  |  |
| - **Long-standing persistent (%)** | 16 (18) | 10 (16) | 5 (19) | 0.76 |  |
| - **Persistent (%)** | 73 (82) | 53 (84) | 21 (81) |  |  |
| **Time since AF diagnosis (yrs)** | 3.0 (1.0, 12.6) | 3.0 (1.0, 12.9) | 3.0 (1.0, 11.3) | 0.63 |  |
| **Female/Male (%)** | 22/67 (25, 75) | 13/50 (21, 79) | 9/17 (35, 65) | 0.16 |  |
| **LVEF (Simpson method, in %)** | 55 (25, 65) | 55 (25, 65) | 55 (25, 65) | 0.98 |  |
| **Left atrial volume (ml)** | 117 (70, 191) | 113 (70, 169) | 122 (73, 196) | 0.36 |  |
| **LAVI (ml/m^2^)** | 56 (35, 88) | 56 (35, 85) | 55 (35, 85) | 0.46 |  |
| **BMI (Kg/m2)** | 28.0 (21.4, 39.0) | 29.0 (21.1, 35.9) | 28.0 (23.0, 42.8) | 0.27 |  |
| **Hypertension (%)** | 6 (6.7) | 6 (9.5) | 0 (0) | 0.17 |  |
| **CAD (%)** | 8 (9.0) | 7 (11) | 1 (3.8) | 0.43 |  |
| **HCM (%)** | 2 (2.2) | 2 (3.2) | 0 (0) | >0.99 |  |
| **VHD (%)** | 9 (10) | 6 (9.5) | 3 (12) | 0.72 |  |
| **OSAS (%)** | 37 (42) | 27 (43) | 10 (38) | 0.66 |  |
| **Diabetes (%)** | 10 (11) | 10 (16) | 0 (0) | 0.031 |  |
| **Tobacco (%)** | 19 (21) | 12 (19) | 7 (27) | 0.41 |  |
| **Hypercholesterolemia (%)** | 31 (35) | 25 (40) | 6 (23) | 0.15 |  |
| **Medication at baseline (index PVI)** |  |  |  |  |  |
| - **Betablocker (%)** | 68 (76) | 50 (79) | 18 (69) | 0.31 |  |
| - **AADs (%)** | 30 (35) | 21 (35) | 9 (36) | 0.93 |  |
| - **CCB (%)** | 7 (7.9) | 3 (4.8) | 4 (15) | 0.19 |  |
| - **ACE inhibitor (%)** | 25 (28) | 18 (29) | 7 (27) | 0.87 |  |
| - **Statin (%)** | 13 (15) | 11 (17) | 2 (7.7) | 0.33 |  |
| - **Angiotensin receptor antagonist (%)** | 11 (12) | 8 (13) | 3 (12) | >0.99 |  |
| **Cumulative ablation time of index procedure (sec)** | 2061 (1241-3532) | 2051 (1237- 3367) | 2071 (1288-4139) | 0.53 |  |

^1^Median and IQR (5%-95%) or Frequency (%)

AADs = antiarrhythmics ; ACE = angiotensin converting enzyme ; LAVI = left atrial volume index ; CAD =coronary Artery Disease ; CCB = calcium channel blocker; FU = follow-up ; HCM = hypertrophic cardiomyopathy ; OSAS = obstructive sleep apnea syndrome ; PVI = pulmonary vein isolation ; VHD = valvular heart disease

^2^ Mann–Whitney U-test; Pearson's Chi-squared test; Fisher's exact test

**
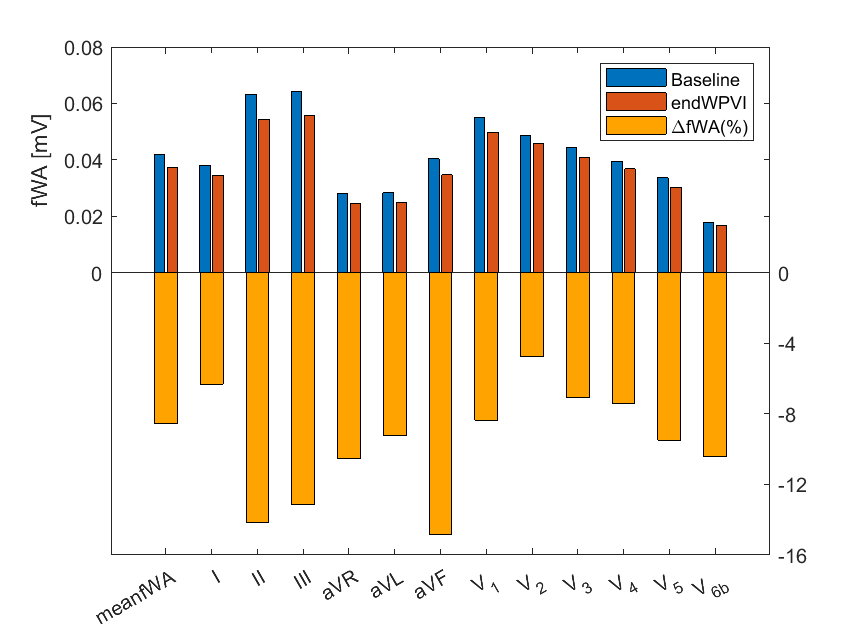
**

**Supplementary Figure S1** **Effect of first time WPVI on f-waves amplitude on the entire study population (n = 114)**

Mean (meanfWA) of the fWA values across the 12 ECG leads, and absolute fWA values at baseline (blue colour) and at endWPVI (red colour) are displayed for each individual ECG lead. Relative change (ΔfWA%) in fWA at endWPVI from the baseline fWA is indicated in orange colour


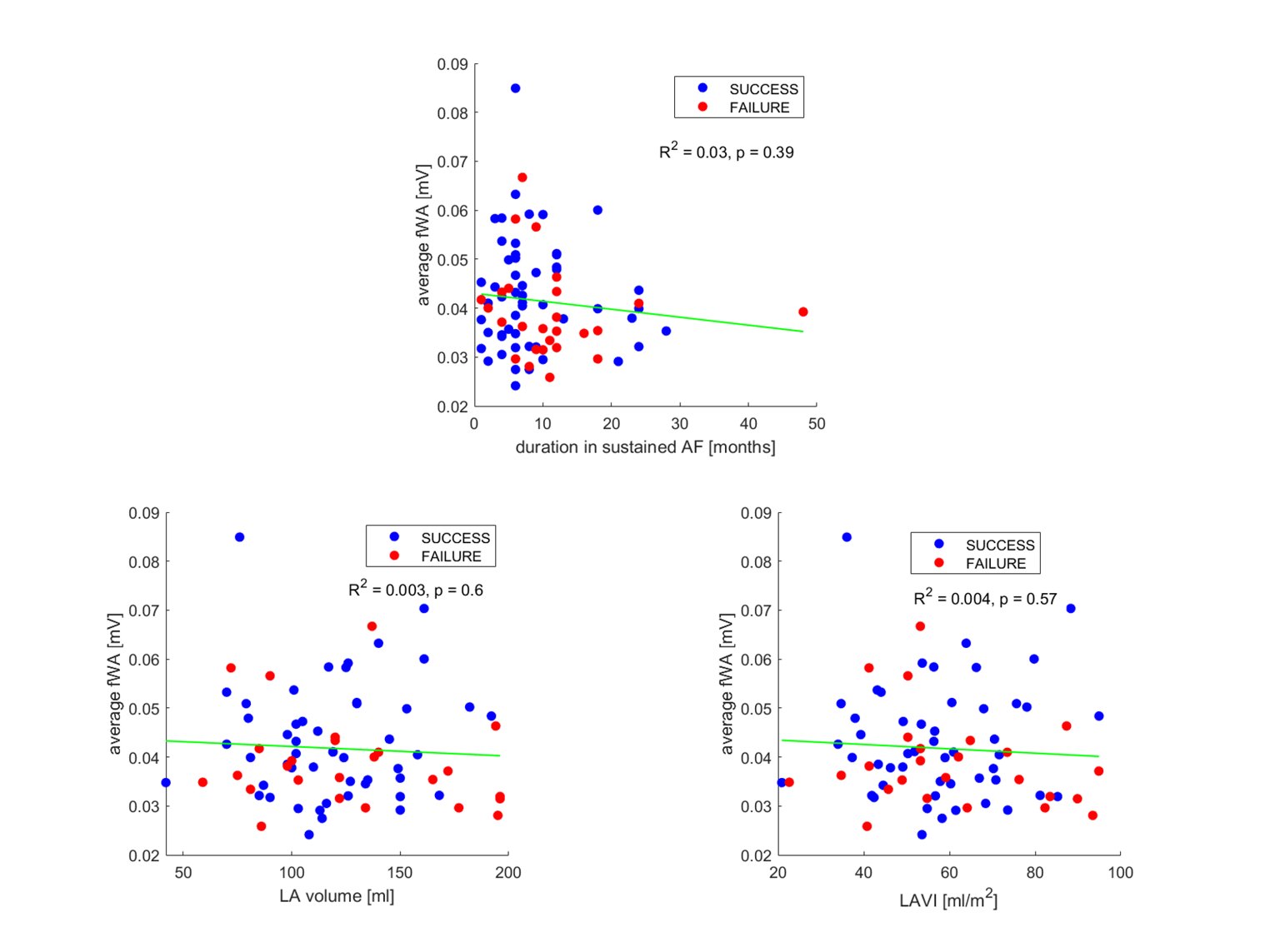


**Supplementary Figure S2 Relationship between the amplitude of f-waves and duration in sustained AF (upper-middle panel), left atrium volume (lower-left panel) and left atrium volume index (lower-right panel).**

**Supplementary Table S2** Amplitude of f-waves at baseline according to long-term ablation outcome

| **ECG lead** | **SUCCESS**  **AF freedom OFF AADs**  **(n = 54 )**^1^ | **FAILURE**  **AF recurrence after redo WPVI**  **(n = 26)**^1^ | **p-value**^2^ |
| --- | --- | --- | --- |
| **I** | 0.033 (0.022, 0.073) | 0.033 (0.025, 0.056) | 0.914 |
| **II** | 0.064 (0.040, 0.094) | 0.055 (0.040, 0.108) | 0.438 |
| **III** | 0.064 (0.040, 0.099) | 0.055 (0.040, 0.100) | 0.132 |
| **AVR** | 0.027 (0.019, 0.041) | 0.024 (0.019, 0.043) | 0.397 |
| **AVL** | 0.026 (0.017, 0.048) | 0.023 (0.020, 0.036) | 0.288 |
| **AVF** | 0.040 (0.025, 0.063) | 0.035 (0.024, 0.071) | 0.261 |
| **V1** | 0.050 (0.032, 0.098) | 0.044 (0.030, 0.083) | **0.046** |
| **V2** | 0.047 (0.032, 0.074) | 0.046 (0.029, 0.068) | 0.374 |
| **V3** | 0.044 (0.030, 0.073) | 0.041 (0.028, 0.058) | 0.122 |
| **V4** | 0.041 (0.025, 0.065) | 0.036 (0.025, 0.053) | **0.024** |
| **V5** | 0.034 (0.021, 0.055) | 0.029 (0.022, 0.050) | **0.026** |
| **V6** | 0.017 (0.011, 0.030) | 0.016 (0.011, 0.028) | 0.391 |
| **meanfWA** | 0.041 (0.029, 0.061) | 0.037 (0.028, 0.058) | 0.112 |
| ^1^Median (IQR); ^2^Wilcoxon rank sum test | | | |

**Supplementary Table S3** Amplitude of f-waves at the end of WPVI according to long-term ablation outcome

| **ECG lead** | **SUCCESS**  **AF freedom OFF AADs**  **(n = 54 )**^1^ | **FAILURE**  **AF recurrence after redo WPVI**  **(n = 26)**^1^ | **p-value**^2^ |
| --- | --- | --- | --- |
| **I** | 0.034 (0.018, 0.059) | 0.028 (0.019, 0.048) | 0.174 |
| **II** | 0.054 (0.031, 0.093) | 0.046 (0.026, 0.088) | 0.077 |
| **III** | 0.057 (0.036, 0.090) | 0.045 (0.029, 0.083) | **0.004** |
| **aVR** | 0.025 (0.015, 0.041) | 0.020 (0.011, 0.035) | 0.075 |
| **aVL** | 0.026 (0.014, 0.041) | 0.020 (0.014, 0.031) | **0.007** |
| **aVF** | 0.032 (0.022, 0.062) | 0.029 (0.017, 0.055) | **0.026** |
| **V1** | 0.046 (0.027, 0.107) | 0.040 (0.023, 0.062) | 0.056 |
| **V2** | 0.046 (0.026, 0.067) | 0.039 (0.022, 0.066) | 0.269 |
| **V3** | 0.040 (0.026, 0.064) | 0.038 (0.023, 0.051) | 0.091 |
| **V4** | 0.036 (0.023, 0.054) | 0.031 (0.021, 0.054) | **0.039** |
| **V5** | 0.029 (0.018, 0.047) | 0.029 (0.018, 0.050) | 0.299 |
| **V6** | 0.016 (0.010, 0.027) | 0.015 (0.008, 0.028) | 0.188 |
| **meanfWA** | 0.039 (0.024, 0.053) | 0.032 (0.020, 0.046) | **0.018** |
| ^1^Median (IQR); ^2^Wilcoxon rank sum test | | | |
